# Synthesis of Amido-Quinoline-Based Hafnium and Zirconium Complexes and Their Catalytic Properties for Ethylene/1-Octene Copolymerization

**DOI:** 10.3390/polym17040449

**Published:** 2025-02-08

**Authors:** Qiqi He, Ruijun Zhang, Junhua Li, Yuexin Hu, Yong Zheng, Jianhua Qian

**Affiliations:** 1 College of Chemistry and Chemical Engineering, China University of Petroleum (East China), Qingdao 266555, China; b21030018@s.upc.edu.cn (Q.H.); zhangruijun523@gmail.com (R.Z.); 2 College of Petrochemical Engineering, Liaoning Petrochemical University, Fushun 113001, China; lijunhua0521@163.com (J.L.); yxhu1981@163.com (Y.H.); 3 Key Laboratory of Inorganic Nonmetallic Crystalline and Energy Conversion Materials, College of Materials and Chemical Engineering, China Three Gorges University, Yichang 443002, China

**Keywords:** non-metallocene, amido-quinoline-based hafnium and zirconium complexes, ethylene-1-octene copolymerization, catalytic activity

## Abstract

The development of polyolefin catalysts plays a pivotal role in driving advancements within the polyolefin industry. In this study, five ligands (L1–L5) and six Hf (Hf 1-5) and Zr (Zr-1) metal complexes with amido-quinoline-based ligands were successfully synthesized by a simple and efficient synthetic route. The new Hf (Hf 1-5) and Zr (Zr-1) complexes exhibit high thermal stability, moderate activity, and excellent 1-octene incorporation capability. As a result, they have been successfully utilized in high-temperature solution-phase polymerization to produce polyolefin elastomers (POEs). The electron-donating effect of the ligand was identified as a crucial factor contributing to the improved catalytic performance and comonomer incorporation capability. The steric effects of substituents on the ligand have little impact on the olefin copolymerization activity, molecular weight, and comonomer incorporation capability. The Hf-1 complex demonstrates outstanding copolymerization activity and comonomer incorporation (8.3 × 10^6^ g polymer/(mol catalyst · h), 26 wt%), offering significant potential for large-scale operations and practical applications.

## 1. Introduction

A polyolefin elastomer (POE) is a random copolymer of ethylene with a high α-olefin content (typically exceeding 20 wt%), synthesized by copolymerizing ethylene with 1-octene, 1-hexene, or 1-butene [1,2,3,4,5]. The high comonomer content in a POE results in a polymer chain comprising crystalline resin and amorphous rubber phases. This unique structure imparts to the material the high elasticity of rubber and the plasticity of thermoplastic resins, enabling easy processing and molding [6,7]. As a versatile material, POEs find a wide range of applications in photovoltaics, automotive components, construction, cables, and more.

**Scheme 1:**
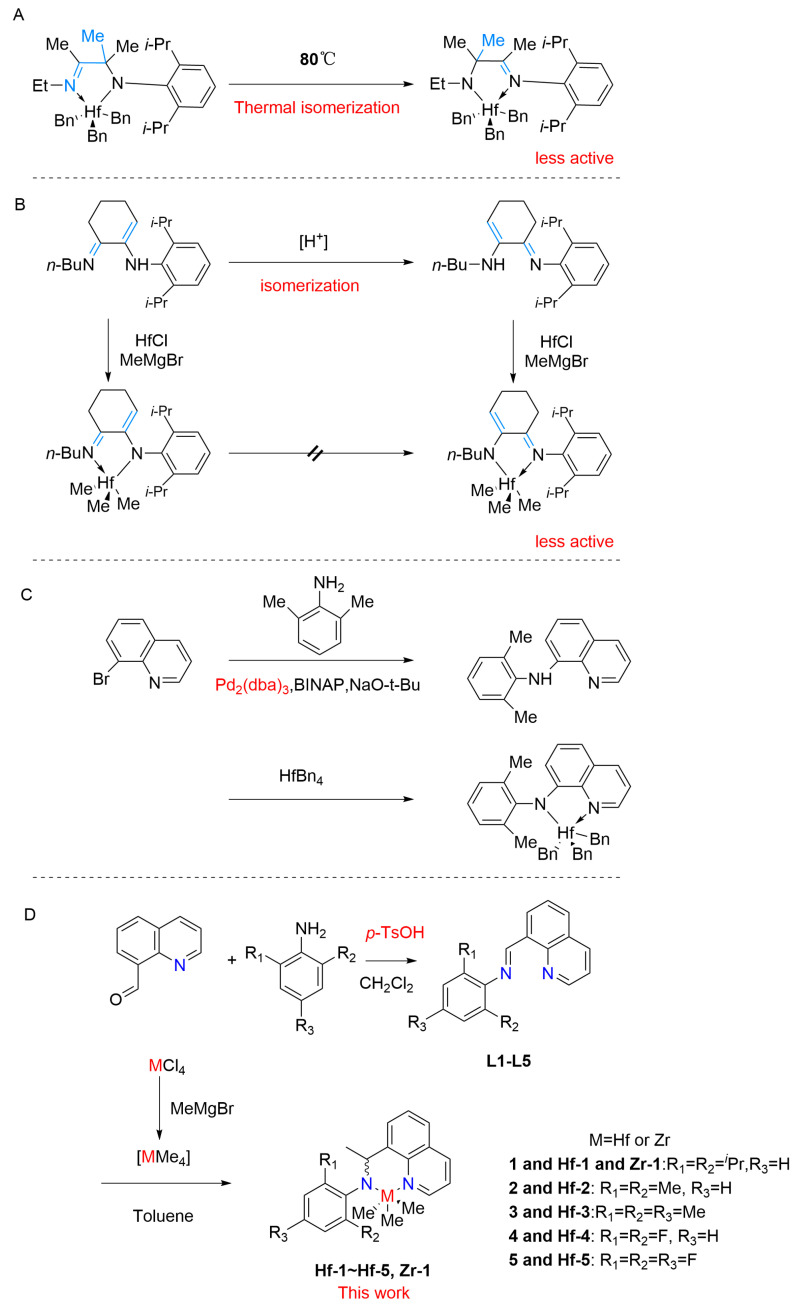
Synthesis of ligands and metal complexes. (**A**) synthesis of imino-amido metal complexes (**B**) synthesis of imino-enamido metal complexes (**C**) synthesis of amido-quinoline metal complexes (**D**) the research presented in this paper.

The industrial production of POEs employs high-temperature solution polymerization [8,9,10,11]. This method ensures that the copolymer does not precipitate during the reaction, maintaining a homogeneous reaction system. As a result, the obtained copolymer exhibits a uniform comonomer distribution and narrow molecular weight distribution. Consequently, the catalyst must possess excellent high-temperature stability to meet the requirements for this process. The catalysts used in industrial applications are typically constrained geometry catalysts (CGCs), which involve complex synthesis routes and high raw material costs [12,13,14,15]. In recent years, non-metallocene catalysts have gained widespread attention due to their simple synthesis and tunable ligand structures [16,17,18,19,20,21,22,23,24,25]. Among them, [NN] bidentate ligand metal complexes have demonstrated excellent high-temperature stability, showing great potential for industrial application. Klosin found that imine-amino Hf catalysts exhibit high polymerization activity and excellent copolymerization performance. However, further studies have shown that these catalysts are thermodynamically unstable and tend to undergo 1,2-methyl rearrangement to form more thermodynamically stable metal complexes. The polymerization activity of these transformed metal complexes is significantly reduced (Scheme 1A) [26,27]. These researchers designed a more thermodynamically stable imine-ene-amino Hf catalyst to address this issue using DFT calculations. This metal complex does not undergo rearrangement at high temperatures. However, further studies revealed that although the metal complex does not undergo rearrangement, its ligands undergo rearrangement under acidic conditions, resulting in a new metal complex with significantly reduced polymerization activity (Scheme 1B) [28,29]. The quinoline-amino catalyst was subsequently further developed. This catalyst features a simple synthesis route, and the resulting metal complexes are relatively stable. However, an important issue is that the synthesis process involves an expensive metal Pd catalyst, limiting its potential for large-scale industrial production (Scheme 1C) [30,31,32].

In this study, a novel series of imino-quinoline ligands were designed and synthesized through p-toluenesulfonic acid (p-TsOH)-catalyzed reactions between 8-aldehyde-quinoline and various anilines. Subsequently, amido-quinoline complexes were efficiently prepared in moderate yields through one-pot reactions of imino-quinoline ligands with in situ-formed MMe_4_ (M = Hf, Zr) (Scheme 1D). This method presents a cost-effective route for synthesizing highly thermally and chemically stable amido-quinoline complexes, offering significant potential for large-scale operations and practical applications.

## 2. Experimental Section

### 2.1. Experimental Materials and Characterization Methods

General considerations: All moisture- and oxygen-sensitive reactions and compounds were performed and synthesized using standard Schlenk techniques or glovebox techniques in an atmosphere of high-purity nitrogen. Toluene, dichloromethane, cyclohexane, and 1-octene from Sigma-Aldrich (St. Louis, MO, USA) were dried by refluxing over sodium under nitrogen. C_6_D_6_ from Sigma-Aldrich (St. Louis, MO, USA) was stored over a Na/K alloy in vacuo and vacuum-transferred immediately before use. Ethylene from Shenyang Shuntai Special Gas Co., Ltd. (Shenyang City, China), was purified by an activated 4A molecular sieve column. The MAO solution (10 wt% in toluene) was purchased from Sigma-Aldrich (St. Louis, MO, USA) and was used without further purification. The borate [Ph_3_C][B(C_6_F_5_)_4_] was purchased from Energy Chemical (Anqing City, China). All other chemicals were purchased from commercial suppliers and used without further purification unless otherwise noted.

The structure of the ligand was characterized using an INVENIO-S Fourier Transform Infrared (FTIR) spectrometer (Bruker, Karlsruhe, Germany). The analysis was performed in a wavelength range of 2500~25,000 nm with a resolution of 4 cm⁻¹. ^1^H NMR spectra were recorded on a Bruker Avance NEO 400 MHz NMR spectrometer (400 MHz for ^1^H NMR) (Bruker, Karlsruhe, Germany) equipped with a 5 mm BBO probe. HT-^13^C NMR of polymer microstructures was conducted in a 10% deuterated ortho-dichlorobenzene (o-DCB) solution at 150 °C using a JNM-ECZL400S NMR spectrometer (JEOL, Tokyo, Japan) at 120 °C with a delay time of 5 s. Signals were assigned according to the literature for these polymers. High-temperature gel permeation chromatography (HT-GPC) was carried out in 1,2,4-trichlorobenzene (stabilized with 125 ppm of BHT) at 150 °C with a GPC-IR instrument (Polymer Char, Valencia, Spain) equipped with a set of three Agilent Technologies PLgel 20 µm MIXED-A columns (300 × 7.5 mm) with infrared 6 (IR6) and differential viscosity detectors. Data reported were determined through universal calibration relative to polystyrene standards.

### 2.2. Synthesis of Ligand and Metal Complexes

Synthesis of Compound L1: Quinoline-8-carbaldehyde (0.79 g, 5 mmol) was dissolved in dichloromethane or anhydrous ethanol (20 mL). 2,6-diisopropyl aniline (0.89 g, 5 mmol) and p-TsOH (5 mg) were slowly added at room temperature. The reaction was carried out for 6 h. After the reaction, dichloromethane was removed under reduced pressure, and cyclohexane (20 mL) was added to extract the product. Subsequently, the mixture was filtered, and the solvent was removed under reduced pressure. The 8-(2,6-*^i^*Pr_2_-C_6_H_3_-N = CH)-quinoline ligand L1 was obtained as a yellow powder (1.12 g, 83%). ^1^H NMR (400 MHz, benzene-d_6_) δ: 8.89 (dd, J = 7.2, 1.6 Hz, 1H), 8.64 (dd, J = 4.1, 1.8 Hz, 1H), 7.46 (dd, J = 8.3, 1.8 Hz, 1H), 7.39 (dd, J = 8.2, 1.6 Hz, 1H), 7.29–7.21 (m, 3H), 7.21–7.17 (m, 1H), 6.73 (dd, J = 8.3, 4.1 Hz, 1H), 3.35 (hept, J = 6.9 Hz, 2H), and 0.30 (s, 6H). The ^1^H NMR spectrum is shown in Figure S1.

Synthesis of Compound L2: Using the method described for Compound L1, Compound L2 was obtained as a yellow powder (1.13 g, 86%). ^1^H NMR (400 MHz, benzene-d_6_) δ: 8.88 (dd, J = 7.2, 1.6 Hz, 1H), 8.65 (dd, J = 4.2, 1.8 Hz, 1H), 7.48 (dd, J = 8.3, 1.8 Hz, 1H), 7.39 (dd, J = 8.1, 1.5 Hz, 1H), 7.25 (ddd, J = 8.1, 7.1, 0.7 Hz, 1H), 7.07 (dq, J = 7.1, 0.8 Hz, 2H), 6.99 (dd, J = 8.3, 6.6 Hz, 1H), 6.75 (dd, J = 8.3, 4.1 Hz, 1H), and 2.25 (s, 6H). The ^1^H NMR spectrum is shown in Figure S2.

Synthesis of Compound L3: Using the method described for Compound L1, Compound L3 was obtained as a yellow powder (1.21 g, 88%). ^1^H NMR (400 MHz, benzene-d_6_) δ: 8.88 (dd, J = 7.2, 1.6 Hz, 1H), 8.65 (dd, J = 4.2, 1.8 Hz, 1H), 7.48 (dd, J = 8.3, 1.8 Hz, 1H), 7.39 (dd, J = 8.1, 1.5 Hz, 1H), 7.25 (ddd, J = 8.1, 7.1, 0.7 Hz, 1H), 7.07 (dq, J = 7.1, 0.8 Hz, 2H), 6.99 (dd, J = 8.3, 6.6 Hz, 1H), 6.75 (dd, J = 8.3, 4.1 Hz, 1H), and 2.25 (s, 9H).

Synthesis of Compound L4: Quinoline-8-carbaldehyde (0.79 g, 5 mmol) was dissolved in dichloromethane (20 mL). 2,6-difluoroaniline (0.65 g, 5 mmol) and p-toluenesulfonic acid (5 mg) were slowly added at room temperature. The reaction was carried out for 6 h. After the reaction, dichloromethane was removed under reduced pressure, and anhydrous methanol (20 mL) was then added to extract the product. The 8-(2,6-F_2_-C_6_H_3_-N = CH)-quinoline ligand L4 was filtered to obtain it as a yellow powder (0.85 g, 63%). ^1^H NMR (400 MHz, benzene-d_6_) δ: 8.87 (dd, J = 7.3, 1.5 Hz, 1H), 8.63 (dd, J = 4.1, 1.8 Hz, 1H), 7.42 (dd, J = 8.3, 1.8 Hz, 1H), 7.33 (dd, J = 8.1, 1.5 Hz, 1H), 6.71 (dd, J = 8.3, 4.1 Hz, 1H), 6.65–6.57 (m, 2H), and 6.50–6.41 (m, 1H). The ^1^H NMR spectrum is shown in Figure S3.

Synthesis of Compound L5: Using the method described for Compound L4, Compound L5 was obtained as a yellow powder (0.92 g, 64%). ^1^H NMR (400 MHz, benzene-d_6_) δ: 8.84 (dd, J = 7.3, 1.5 Hz, 1H), 8.65 (dd, J = 4.1, 1.8 Hz, 1H), 7.43 (dd, J = 8.3, 1.8 Hz, 1H), 7.34 (dd, J = 8.1, 1.5 Hz, 1H), 6.72 (dd, J = 8.3, 4.1 Hz, 1H), and 6.34–6.21 (m, 2H). The ^1^H NMR spectrum is shown in Figure S4.

Synthesis Method 1 for Hf-1: Hafnium tetrachloride (0.64 g, 2 mmol) was dissolved in anhydrous toluene (20 mL). Under −40 °C conditions, methylmagnesium bromide solution (2.7 mL, 3 mol/L in ether) was slowly added and stirred for 2 h. Then, the L1 ligand (0.52 g, 1.8 mmol) was added, and the reaction was continued at −40 °C for another 2 h. Subsequently, the reaction was continued at room temperature for another 6 h. After the reaction, the solvent was removed under reduced pressure, and dichloromethane (20 mL) was added. The reaction mixture was stirred for 5 min and filtered to obtain a red-brown filtrate. The filtrate was concentrated to yield Hf-1 (0.40 g, 40%). ^1^H NMR (400 MHz, benzene-d_6_) δ: 8.51 (dd, J = 4.1, 1.9 Hz, 1H), 7.42 (dd, J = 8.3, 1.9 Hz, 1H), 7.29 (dd, J = 7.0, 1.5 Hz, 1H), 7.18–7.15 (m, 1H), 7.03 (s, 2H), 6.95–6.88 (m, 2H), 6.64 (dd, J = 8.3, 4.1 Hz, 1H), 5.28 (d, J = 10.6 Hz, 1H), 4.88 (q, J = 6.9 Hz, 1H), 3.48 (p, J = 6.8 Hz, 2H), 1.78 (d, J = 6.8 Hz, 3H), 1.16 (d, J = 6.9 Hz, 6H), 1.02 (d, J = 6.8 Hz, 6H), and 0.18 (s, 9H). The ^1^H NMR spectrum is shown in Figure S5.

Synthesis Method 2 for Hf-1. Hafnium tetrachloride (0.64 g, 2 mmol) and 8-(2,6-*^i^*Pr₂-C₆H₃-N = CH)-quinoline ligand L1 (0.57 g, 1.8 mmol) were dissolved in anhydrous toluene (20 mL). At room temperature, methylmagnesium bromide solution (2.7 mL, 3 mol/L in ether) was slowly added and stirred for 3 h. The suspension turned deep yellow and then became black within a few minutes. After the reaction, the solvent was removed under reduced pressure, and dichloromethane (20 mL) was added. The reaction mixture was stirred for 5 min and filtered to obtain a red-brown filtrate. The filtrate was concentrated to yield Hf-1 (0.42 g, 42%). ^1^H NMR (400 MHz, benzene-d_6_) δ: 8.51 (dd, J = 4.1, 1.9 Hz, 1H), 7.42 (dd, J = 8.3, 1.9 Hz, 1H), 7.29 (dd, J = 7.0, 1.5 Hz, 1H), 7.18–7.15 (m, 1H), 7.03 (s, 2H), 6.95–6.88 (m, 2H), 6.64 (dd, J = 8.3, 4.1 Hz, 1H), 5.28 (d, J = 10.6 Hz, 1H), 4.88 (q, J = 6.9 Hz, 1H), 3.48 (p, J = 6.8 Hz, 2H), 1.78 (d, J = 6.8 Hz, 3H), 1.16 (d, J = 6.9 Hz, 6H), 1.02 (d, J = 6.8 Hz, 6H), and 0.18 (s, 9H).

Using Method 2 described for Hf-1, Hf 2-5 and Zr-1 were obtained using a similar approach.

### 2.3. Ethylene/1-Octene Copolymerization Experiment

At room temperature, cyclohexane (100 mL) and an appropriate amount of 1-octene were added to a 250 mL stainless steel reactor pre-evacuated and purged with nitrogen. Mechanical stirring was started at 600 rpm, and heating was begun. When the temperature reached the reaction temperature, ethylene was introduced to increase the pressure to 3.0 MPa. Once the set pressure was reached, the reactor was pre-saturated for 5 min. Then, a dichloromethane solution (10 mL) containing a catalyst and cocatalyst [Ph_3_C][B(C_6_F_5_)_4_] was introduced into the reactor using a syringe to initiate the polymerization reaction.

During the reaction, a constant reaction temperature, ethylene pressure, and stirring rate were maintained. After 15 min, the reaction was terminated by adding a 5 wt% hydrochloric acid ethanol solution. The copolymer was isolated and washed several times with ethanol. The copolymer was vacuum-dried at 60 °C to a constant weight.

The formula for calculating catalyst activity (Act.) is

(1) $$\mathrm{Act}.=\frac{m_{\mathrm{polymer}}}{mol_{\mathrm{Cat}}\cdot h}$$

where *m*_polymer_ is the mass of the copolymer in units of g, *mol*_cat_ is the molar amount of the catalyst in units of mol, *h* is the copolymerization reaction time in units of h, and Act. is the copolymerization activity in units of g_polymer_/(mol_cat_ ·h).

## 3. Results and Discussion

### 3.1. Analysis of Synthesis Conditions for Ligands

In this study, five novel quinoline-imine ligands were successfully synthesized through the reaction of quinoline-8-carbaldehyde with substituted anilines. The reaction was carried out at room temperature. When anhydrous ethanol was used as the solvent, the L1 ligand precipitated directly from the solution, with an overall yield of approximately 70%. However, when anhydrous dichloromethane was used as the solvent, the L1 ligand was completely soluble, and the reaction yield increased to over 83%. As a result, dichloromethane was selected as the solvent for subsequent reactions.

After the reaction, the solvent was removed under reduced pressure. The experimental results show that quinoline-8-carbaldehyde is insoluble in cyclohexane but soluble in methanol. The ligands were dissolved in cyclohexane and filtered for the L1, L2, and L3 ligands, and the filtrate was concentrated to obtain pure ligands, with yields exceeding 80%. In contrast, the L4 ligand and L5 ligand exhibited limited solubility in cyclohexane. Anhydrous methanol was therefore used to dissolve the reaction products, and the ligands were obtained directly after filtration, with yields of 63% and 64%, respectively. These lower yields were primarily attributed to the partial solubility of the L4 and L5 ligands in anhydrous methanol.

**Figure 1:**
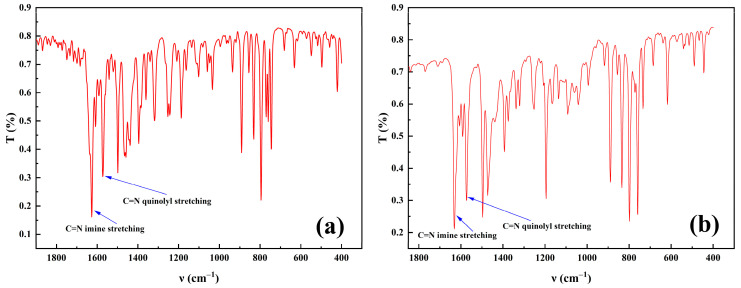
(**a**) Infrared spectrum of ligand L1; (**b**) infrared spectrum of ligand L2.

The infrared spectra of the L1 and L2 ligands are shown in Figure 1. The peak at 1625 cm⁻¹ corresponds to the stretching vibration of the C = N imine group, while the peak at 1575 cm⁻¹ corresponds to the stretching vibration of the C = N quinolyl group. No significant strong absorption peaks were observed in the 1900-1650 cm⁻¹ range, indicating the disappearance of the C = O bond and suggesting that the ligands were successfully synthesized.

### 3.2. Analysis of Synthesis Conditions for Metal Complexes

This study explored two synthesis methods for metal complexes using the L1 ligand. In Method 1, hafnium tetrachloride and methylmagnesium bromide were added to toluene (20 mL) and reacted at −40 °C for 2 h. Subsequently, a toluene solution (10 mL) containing the L1 ligand was added, followed by another 2 h reaction at low temperature and 6 h reaction at room temperature. In Method 2, a toluene solution of the ligand and hafnium tetrachloride was prepared at room temperature. Then, a methylmagnesium bromide solution was added dropwise, reacting for 3 h at room temperature. During both reactions, the solution initially turned yellow. In Method 1, the solution gradually darkened upon returning to room temperature, while in Method 2, it began to darken within a few minutes after adding methylmagnesium bromide. This is a typical characteristic of this type of reaction [29]. After the reactions, the solvent was removed under reduced pressure, and dichloromethane (20 mL) was added for extraction. After stirring for 5 min and filtering, the filtrate was concentrated to yield a red-brown powder. The Method 1 and Method 2 yields were 40% and 42%, respectively. Although the yields were similar, Method 2 was more time-efficient and did not require low-temperature conditions, making it the preferred method for subsequent complexation reactions.

**Figure 2:**
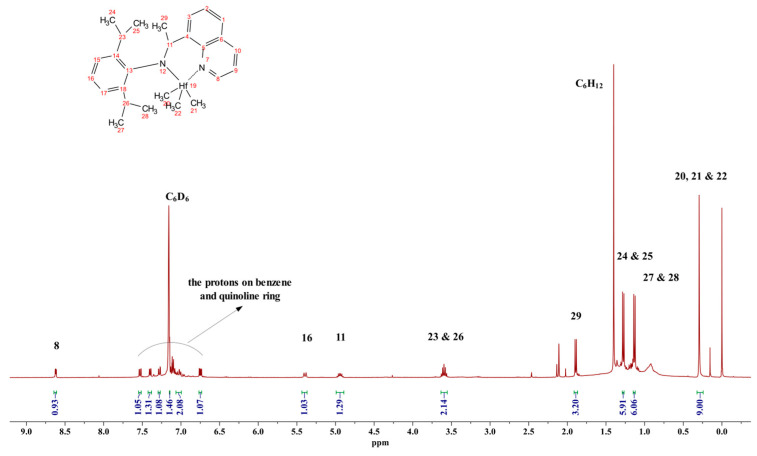
The ^1^H NMR spectrum of the metal complex Hf-1.

The ^1^H NMR spectrum of the metal complex Hf-1 is shown in Figure 2. As shown in Figure 2, the single resonance peak at 0.3 ppm is assigned to (CH_3_)_3_Hf, the resonance at 1.2–1.5 ppm corresponds to the hydrogens in the isopropyl group, the resonance peak at 1.4 ppm corresponds to the peak of impurity for cyclohexane hydrogens in deuterated benzene, the resonance peak at 3.52 ppm corresponds to the β-hydrogens in the isopropyl group, and the resonance peaks between 6.5 and 7.5 ppm correspond to the hydrogens in the quinoline ring. Notably, a quartet resonance at 4.95 ppm was observed and assigned to CH_3_CHN, while the resonance at 1.8 ppm was assigned to CH_3_CHN. This result suggested the migration of methyl and the formation of the amino group, similarly to previous reports involving imine-type ligands [24,33].

### 3.3. Ethylene/1-Octene Copolymerization Performance

Table 1 summarizes the catalytic results of ethylene/1-octene copolymerization using Hf and Zr metal complexes with different ligand structures. These copolymers were synthesized under identical conditions, with the only variation being the ligand structure.

**Figure 3:**
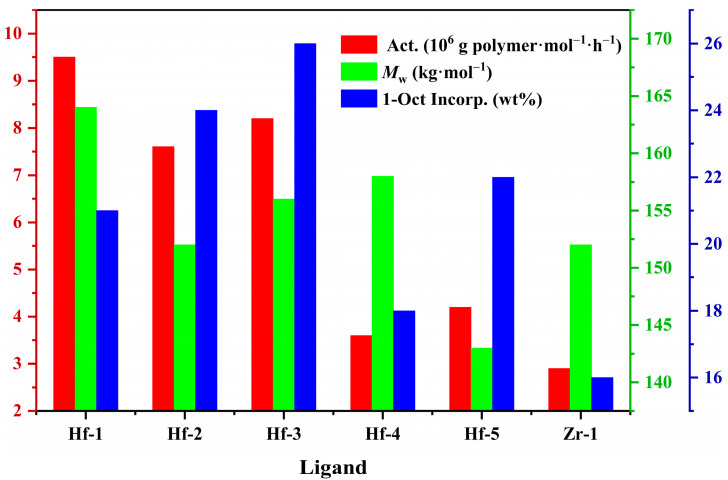
The effect of ligand and metal center types on copolymerization reaction activity, molecular weight, and comonomer incorporation.

The structure of the ligand determines the properties of the metal complex, which in turn significantly affects the catalytic activity of the polymerization reaction. The effect of ligand and metal center types on polymerization reaction activity, molecular weight, and comonomer incorporation are shown in Figure 3. As shown in Figure 3, the metal complexes Hf-4 and Hf-5, containing fluorine (F) substituents, which are typical electron-withdrawing groups, exhibit lower catalytic activity (4.2 and 3.6 × 10^6^ g polymer/(mol catalyst ·h)) and lower comonomer incorporation (18 and 22 wt%) compared to Hf-1 (8.3 × 10^6^ g polymer/(mol catalyst ·h), 26 wt%). This indicates that the electronic effect of substituents on the ligand significantly influences the olefin polymerization activity and comonomer incorporation. In contrast, the steric effects of substituents on the ligand have little impact on the olefin copolymerization activity and comonomer incorporation. Larger steric hindrance helps suppress β-elimination and chain transfer reactions. As a result, the Hf-1 metal complex exhibits the largest molecular weight (164 kg/mol) compared to the other metal complexes. The nature of the metal center has a significant impact on the olefin polymerization activity. The reaction activity and comonomer incorporation of the Hf-1 metal complex (8.3 × 10^6^ g polymer/(mol catalyst ·h)) are significantly higher than those of the Zr-1 metal complex (2.9 × 10^6^ g polymer/(mol catalyst ·h)).

The metal complex’s activity directly affects the polymerization reaction rate and the chain growth rate. Chain transfer and other side reactions also influence the copolymer’s molecular weight. The catalyst’s varying ligand structures affect electron donation and steric hindrance to the metal center, which in turn alter the probability of chain transfer and side reactions. The interplay of these factors leads to the absence of a direct correlation between copolymerization activity and copolymer molecular weight.

Table 2 summarizes the catalytic performance of the Hf-1 metal complex for ethylene/1-octene copolymerization under different polymerization conditions. All polymerization reactions were conducted under an ethylene pressure of 3 MPa and a reaction time of 15 min.

**Figure 4:**
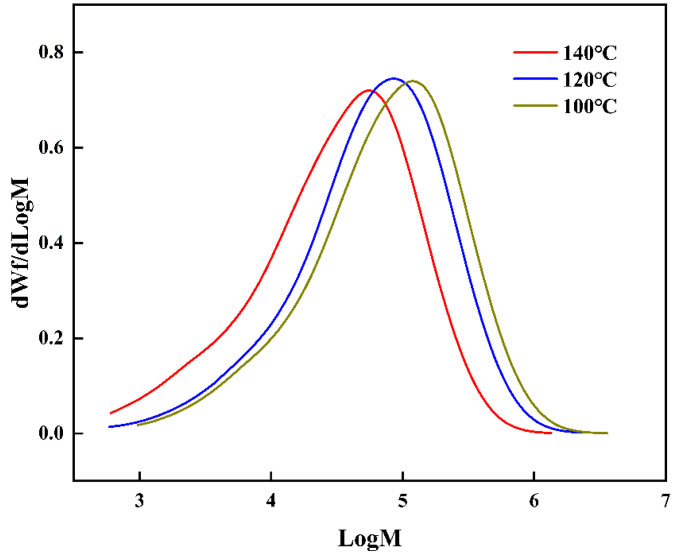
The effect of temperature on the molecular weight of copolymers for Hf-1.

**Figure 5:**
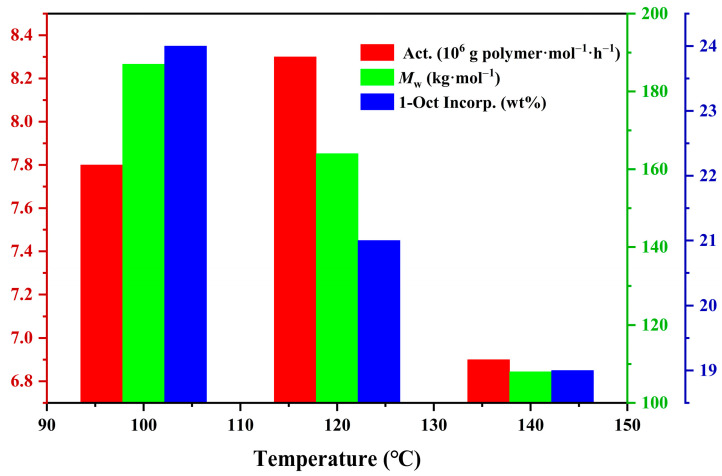
The effect of temperature on copolymerization reaction activity, molecular weight, and comonomer incorporation by Hf-1.

The copolymerization temperature significantly impacts olefin polymerization activity, copolymer molecular weight, and comonomer incorporation. As shown in Figure 4 and Figure 5, the molecular weight of the copolymer gradually decreases with increasing temperature, and the molecular weight distribution broadens. As the reaction temperature increases, the copolymerization activity first increases and then decreases. In the 100–120 °C range, increasing temperature favors the rapid formation of catalytic active sites, significantly increasing the chain growth reaction rate. However, as the temperature continues to rise, the β-H elimination and chain transfer reaction rates also accelerate significantly. The combined effect of both reactions leads to a decrease in both catalytic activity and copolymer molecular weight.

**Figure 6:**
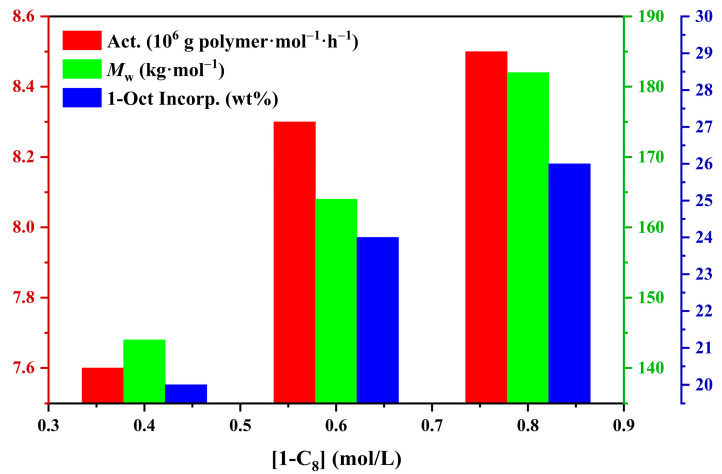
The effect of 1-octene molar concentration on copolymerization reaction activity, molecular weight, and comonomer incorporation by Hf-1.

The effect of 1-octene molar concentration on copolymerization reaction activity, molecular weight, and comonomer incorporation by Hf-1 is shown in Figure 6. The initial amount of 1-octene feed is shown in Table S2. As the molar concentration of the comonomer increases, copolymerization activity gradually increases, exhibiting a typical comonomer effect [36,37]. The molecular weight of the copolymer and comonomer incorporation also gradually increase. However, when the comonomer molar concentration reaches 0.8 M, comonomer incorporation no longer increases significantly.

**Figure 7:**
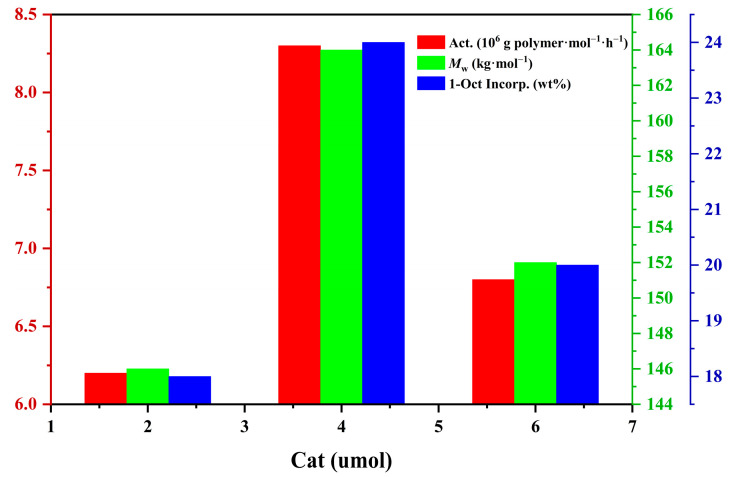
The effect of catalyst dosage on copolymerization reaction activity, molecular weight, and comonomer incorporation by Hf-1.

The effect of catalyst dosage on copolymerization reaction activity, molecular weight, and comonomer incorporation by Hf-1 is shown in Figure 7. The olefin copolymerization activity shows a trend of increasing first and then decreasing with the increase in catalyst amount. When the catalyst amount is small, the number of active sites in the polymerization reaction is limited, and increasing the catalyst amount further improves the catalytic activity. However, when the catalyst amount reaches 6 μmol, the polymerization activity decreases to 6.8 × 10^6^ g polymer/(mol catalyst ·h), and the molecular weight of the obtained copolymer drops to 152 kg/mol. This indicates that when the catalyst concentration is too high, the metal active centers aggregate, leading to a rapid increase in molecular weight, which causes entanglement between copolymer chains and suppresses the chain growth reaction rate, resulting in a decrease in both catalytic activity and copolymer molecular weight.

**Figure 8:**
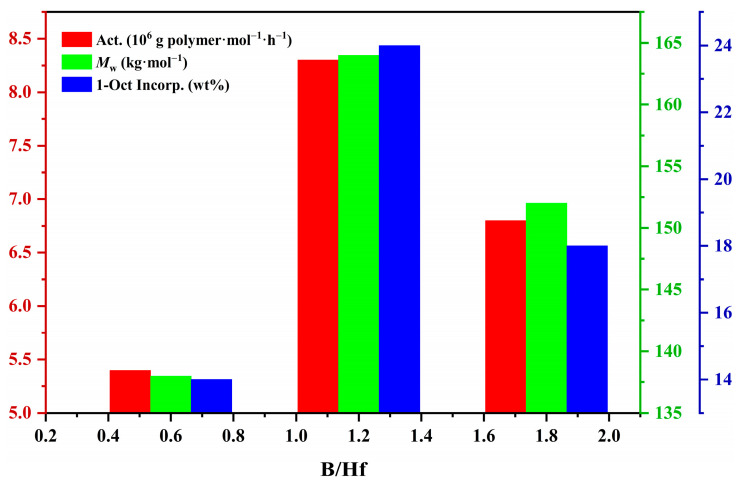
The effect of cocatalyst dosage on copolymerization reaction activity, molecular weight, and comonomer incorporation by Hf-1.

The effect of cocatalyst dosage on copolymerization reaction activity, molecular weight, and comonomer incorporation by Hf-1 is shown in Figure 8. The amount of cocatalyst significantly affects olefin polymerization activity and the properties of the copolymer. In the low concentration range, increasing the amount of cocatalyst enhances polymerization activity and increases the molecular weight of the copolymer. However, as the amount of cocatalyst continues to increase, both copolymerization activity and molecular weight decrease. This is because when the amount of the cocatalyst is too high, the rate of chain transfer reactions in the copolymer chains increases significantly, thereby reducing both the reaction activity and copolymer molecular weight.

## 4. Conclusions

In this study, five ligands and six Hf (Hf 1-5) and Zr (Zr-1) metal complexes with imino-quinoline ligands were successfully synthesized and structurally characterized by FTIR and ^1^H NMR spectroscopy. The findings indicate that the metal center and ligand structure significantly influence copolymerization activity and comonomer incorporation capability. The Hf-1 complex exhibited an exceptionally high catalytic activity and comonomer incorporation capability (8.3 × 10^6^ g polymer/(mol catalyst · h), 26 wt%), outperforming the Zr complex with the same ligand. The electron-donating effect of the ligand was identified as a crucial factor contributing to the improved catalytic performance and comonomer incorporation. The steric effects of substituents on the ligand have little impact on the olefin copolymerization activity, molecular weight, and comonomer incorporation capability. Furthermore, synthesizing these Hf and Zr metal complexes involved the use of inexpensive and readily available raw materials, followed a simple and efficient synthetic route, and resulted in high yields. The catalysts developed in this study demonstrate significant potential for application in industrial polyolefin production.

## Figures and Tables

**Table 1:** Ethylene/1-octene copolymerization results in different ligands of catalysts ^a^.

Run	Cat.	Yield (g)	Act. ^b^	*M*_w_ ^c^	*Ð*	1-Octene Incorp. (wt%) ^d^
1	Hf-1	8.3	8.3	164	2.6	26
2	Hf-2	7.6	7.6	152	2.4	24
3	Hf-3	7.2	7.2	148	2.4	21
4	Hf-4	4.2	4.2	158	2.3	18
5	Hf-5	3.6	3.6	143	2.2	22
6	Zr-1	2.9	2.9	152	2.5	16

^a^ Reaction conditions: *T* = 120 °C; *P* = 3 MPa; [1-C_8_] = 0.6 mol·L^−1^; Cat = 4 umol; cocatalyst [Ph_3_C][B(C_6_F_5_)_4_] = 4.8 umol (B/Hf = 1.2); MAO = 0.2 mL (as scavenger); cyclohexane = 100 mL; polymerization time = 15 min. ^b^ Activity is in units of 10^6^ g polymer/(mol catalyst ·h). ^c^ *M*_w_ is in units of kg/mol. ^d^ The polymers’ comonomer content (wt%) was determined by HT-^13^C NMR [34,35], the HT-^13^C NMR spectra of the copolymers are shown in Figures S6–S11, the chemical shift assignments of the HT-^13^C NMR spectra are provided in Table S1.

**Table 2:** Ethylene/1-octene copolymerization results in different reaction conditions for Hf-1.

Run	Cat.	*T* (°C)	[1-C_8_] (M)	Cat (umol)	B/Hf	Yield (g)	Act.	*M*_w_	*Ð*	1-Octene Incorp. (wt%)
1	Hf-1	100	0.6	4	1.2	7.8	7.8	187	2.4	28
2	Hf-1	120	0.6	4	1.2	8.3	8.3	164	2.6	26
3	Hf-1	140	0.6	4	1.2	6.9	6.9	108	3.2	21
4	Hf-1	120	0.4	4	1.2	7.6	7.6	144	2.8	20
5	Hf-1	120	0.8	4	1.2	8.5	8.5	182	3.0	27
6	Hf-1	120	0.6	2	1.2	6.2	6.2	146	2.4	18
7	Hf-1	120	0.6	6	1.2	6.8	6.8	152	2.8	20
8	Hf-1	120	0.6	4	0.6	5.4	5.4	138	2.3	14
9	Hf-1	120	0.6	4	1.8	6.8	6.8	152	2.8	22

## Data Availability

The relevant data generated and analyzed in the current study are contained within the article and Supplementary Materials. Further inquiries available from the corresponding author upon reasonable request.

## Supplementary Materials

The following supporting information can be downloaded at https://www.mdpi.com/article/10.3390/polym17040449/s1: Figure S1: ^1^H NMR spectrum of L1 in C_6_D_6_; Figure S2: ^1^H NMR spectrum of L2 in C_6_D_6_; Figure S3: ^1^H NMR spectrum of L4 in C_6_D_6_; Figure S4: ^1^H NMR spectrum of L5 in C_6_D_6_; Figure S5: ^1^H NMR spectrum of Hf-1 in C_6_D_6_; Figure S6: HT-^13^C NMR spectrum of the ethylene/1-octene copolymer produced by Hf-1. Figure S7: HT-^13^C NMR spectrum of the ethylene/1-octene copolymer produced by Hf-2. Figure S8: HT-^13^C NMR spectrum of the ethylene/1-octene copolymer produced by Hf-3. Figure S9: HT-^13^C NMR spectrum of the ethylene/1-octene copolymer produced by Hf-4. Figure S10: HT-^13^C NMR spectrum of the ethylene/1-octene copolymer produced by Hf-5. Figure S11: HT-^13^C NMR spectrum of the ethylene/1-octene copolymer produced by Zr-1. Table S1: Assignment of chemical shift regions in the NMR spectrum. Table S2: The initial amount of 1-octene feed.

## References

[1] Sun M., Xiao Y., Liu K., Yang X., Liu P., Jie S., Hu J., Shi S., Wang Q., Lim K.H. (2023). Synthesis and characterization of polyolefin thermoplastic elastomers: A review. Can. J. Chem. Eng..

[2] Mohite A.S., Rajpurkar Y.D., More A.P. (2022). Bridging the gap between rubbers and plastics: A review on thermoplastic polyolefin elastomers. Polym. Bull..

[3] Atiqullah M., Abdelaal A.F., Adamu S., Alasiri H.S. (2024). Catalytic making of ethylene-1-hexene elastomers: Thermodynamic guide to process development. AIChE J..

[4] He Q., Zhang R., Hu Y., Li J., Yu H., Zheng Y., Qian J. (2024). The Effect of Feeding Sequence on the Structure and Properties of the Ethylene/1-Octene Copolymer in the Semi-Continuous Polymerization Reaction System. Polymers.

[5] He Q., Zhang R., Hu Y., Li J., Qian J. (2024). Study on the structure and property of metallocene ethylene/1-heptene and ethylene/1-octene copolymers. J. Macromol. Sci. A Pure Appl. Chem..

[6] Ostoja Starzewski A., Steinhauser N., Xin B.S. (2008). Decisive progress in metallocene-catalyzed elastomer synthesis. Macromolecules.

[7] Xu G., Xiao Y., Wang W., Li B., Liu P. (2023). Rheological and Mechanical Study of Comb-Branched Polyolefin Elastomers Containing Macromer Residues. Macromolecules.

[8] Braunschweig H., Breitling F. (2006). Constrained geometry complexes—Synthesis and applications. Coord. Chem. Rev..

[9] Alt H.G., Köppl A. (2000). Effect of the nature of metallocene complexes of group IV metals on their performance in catalytic ethylene and propylene polymerization. Chem. Rev..

[10] Kaminsky W. (1998). Highly active metallocene catalysts for olefin polymerization. J. Chem. Soc. Dalton Trans..

[11] Ndiripo A., Joubert D., Pasch H. (2016). Ethylene/1-heptene copolymers as interesting alternatives to 1-octene-based LLDPE: Molecular structure and physical properties. J. Polym. Sci. A Polym. Chem..

[12] Wu C.J., Nayab S., Woo H.Y., Hahn J.S., Lee H.S., Kang S.O., Lee H. (2014). Synthesis and X-ray crystal structure of [Me_2_Si(C_5_Me_2_H_2_)(tBuN)]MCl_2_ (M=Ti, Zr) bearing a 3,4-dimethylcyclopentadienyl ring: Investigation of the substitution effect on the cyclopentadienyl (Cp) ring for catalytic performance in ethylene/1-octene (co)polymerization. Polyhedron.

[13] Han S., Yao E., Qin W., Zhang S., Ma Y. (2012). Binuclear Heteroligated Titanium Catalyst Based on Phenoxyimine Ligands: Synthesis, Characterization, and Ethylene (Co)polymerization. Macromolecules.

[14] Mortazavi S.M.M., Arabi H., Zohuri G., Ahmadjo S., Nekoomanesh M., Ahmadi M. (2010). Copolymerization of ethylene/α-olefins using bis(2-phenylindenyl)zirconium dichloride metallocene catalyst: Structural study of comonomer distribution. Polym. Int..

[15] Unruean P., Apisuk W., Kawabata Y., Murayama T., Kitiyanan B., Nomura K. (2019). Effect of supported MAO cocatalysts in ethylene polymerization and ethylene/1-hexene copolymerization using Cp*TiCl_2_(O-2,6-*^i^*Pr_2_C_6_H_3_) catalyst. Mol. Catal..

[16] Makio H., Terao H., Iwashita A., Fujita T. (2011). FI Catalysts for Olefin Polymerization—A Comprehensive Treatment. Chem. Rev..

[17] Wan D.-W., Chen Z., Gao Y.-S., Shen Q., Sun X.-L., Tang Y. (2013). Synthesis and characterization of tridentate [O−N(H)X] titanium complexes and their applications in olefin polymerization. J. Polym. Sci. A Polym. Chem..

[18] Yao E., Wang J., Chen Z., Ma Y. (2014). Homo- and Heteroligated Salicylaldiminato Titanium Complexes with Different Substituents Ortho to the Phenoxy Oxygens for Ethylene and Ethylene/1-Hexene (Co)polymerization. Macromolecules.

[19] Rishina L.A., Kissin Y.V., Lalayan S.S., Gagieva S.C., Tuskaev V.A., Krasheninnikov V.G. (2017). Polymerization of alkenes with a post-metallocene catalyst containing a titanium complex with an oxyquinolinyl ligand. J. Polym. Sci. A Polym. Chem..

[20] Zanchin G., Bertini F., Vendier L., Ricci G., Lorber C., Leone G. (2019). Copolymerization of ethylene with propylene and higher α-olefins catalyzed by (imido)vanadium(iv) dichloride complexes. Polym. Chem..

[21] Gagieva S.C., Tuskaev V.A., Saracheno D., Buyanovskaya A.G., Smirnova O.V., Zvukova T.M., Sizov A.I., Bulychev B.M. (2021). Ethylene homopolymerization and copolymerization with 1-hexene and 1-octene catalyzed by titanium(IV) dichloride TADDOLate complex activated with MAO. Polym. Bull..

[22] Han B., Liu Y., Feng C., Liu S., Li Z. (2021). Development of Group 4 Metal Complexes Bearing Fused-Ring Amido-Trihydroquinoline Ligands with Improved High-Temperature Catalytic Performance toward Olefin (Co)polymerization. Organometallics.

[23] Fang X.-Y., Qin L., Liu J., Shi H., Sun X.-L., Kuang X., Gao Y., Tang Y. (2023). Synthesis and characterization of oxazoline-amine zirconium complexes for ethylene homo- and co-polymerization catalysis. Mol. Catal..

[24] Feng W., Liu C., Liu S., Li Z. (2023). Hafnium and Zirconium Complexes Bearing ONN-Tridentate Ligands and Their Catalytic Properties toward Olefin Polymerization. Organometallics.

[25] Shou J.K., Li P., Tian W.L., Liu Y., Zhang S.J., Wang F.Z., Tan C. (2024). Steric and temperature effects in unsymmetrical α-diimine nickel-catalyzed ethylene and 1-octene polymerization. Appl. Organomet. Chem..

[26] Froese R.D.J., Jazdzewski B.A., Klosin J., Kuhlman R.L., Theriault C.N., Welsh D.M., Abboud K.A. (2011). Imino-Amido Hf and Zr Complexes: Synthesis, Isomerization, and Olefin Polymerization. Organometallics.

[27] Zhang C., Pan H., Klosin J., Tu S., Jaganathan A., Fontaine P.P. (2015). Synthetic Optimization and Scale-Up of Imino–Amido Hafnium and Zirconium Olefin Polymerization Catalysts. Org. Process Res. Dev..

[28] Figueroa R., Froese R.D., He Y., Klosin J., Theriault C.N., Abboud K.A. (2011). Synthesis of Imino-Enamido Hafnium and Zirconium Complexes: A New Family of Olefin Polymerization Catalysts with Ultrahigh-Molecular-Weight Capabilities. Organometallics.

[29] Fontaine P.P., Figueroa R., McCann S.D., Mort D., Klosin J. (2013). Synthesis and Scale-up of Imino–Enamido Hafnium and Zirconium Olefin Polymerization Catalysts. Organometallics.

[30] Fontaine P.P., Ueligger S., Klosin J., Hazari A., Daller J., Hou J. (2015). Development of Improved Amidoquinoline Polyolefin Catalysts with Ultrahigh Molecular Weight Capacity. Organometallics.

[31] Klosin J., Fontaine P.P., Figueroa R. (2015). Development of Group IV Molecular Catalysts for High Temperature Ethylene-α-Olefin Copolymerization Reactions. Acc. Chem. Res..

[32] Szuromi E., Klosin J., Abboud K.A. (2011). Aminotroponiminato Hafnium and Zirconium Complexes: Synthesis and Ethylene/1-Octene Copolymerization Study. Organometallics.

[33] Tsurugi H., Matsuo Y., Yamagata T., Mashima K. (2004). Intramolecular Benzylation of an Imino Group of Tridentate 2,5-Bis(N-aryliminomethyl)pyrrolyl Ligands Bound to Zirconium and Hafnium Gives Amido-Pyrrolyl Complexes That Catalyze Ethylene Polymerization. Organometallics.

[34] De Pooter M., Smith P.B., Dohrer K.K., Bennett K.F., Meadows M.D., Smith C.G., Schouwenaars H.P., Geerards R.A. (1991). Determination of the composition of common linear low density polyethylene copolymers by 13C-NMR spectroscopy. J. Appl. Polym. Sci..

[35] Hsieh E.T., Randall J.C. (1982). Monomer sequence distributions in ethylene-1-hexene copolymers. Macromolecules.

[36] Awudza J.A.M., Tait P.J.T. (2008). The “comonomer effect” in ethylene/α-olefin copolymerization using homogeneous and silica-supported Cp_2_ZrCl_2_/MAO catalyst systems: Some insights from the kinetics of polymerization, active center studies, and polymerization temperature. J. Polym. Sci. A Polym. Chem..

[37] Jiang B., Liu X., Weng Y., Fu Z., He A., Fan Z. (2019). Mechanistic study on comonomer effect in ethylene/1-hexene copolymerization with TiCl_4_/MgCl_2_ model Ziegler-Natta catalysts. J. Catal..

