# Supplementary material for: Synthesis of Amido-Quinoline-Based Hafnium and Zirconium Complexes and Their Catalytic Properties for Ethylene/1-Octene Copolymerization
Source: Polymers (Basel). 2025 Feb 8;17(4):449. doi: 10.3390/polym17040449 (PMC11859631; doi:10.3390/polym17040449)
Supplement: Supplementary file 1 [file polymers-17-00449-s001.zip › polymers-3465947-supplementary.pdf]

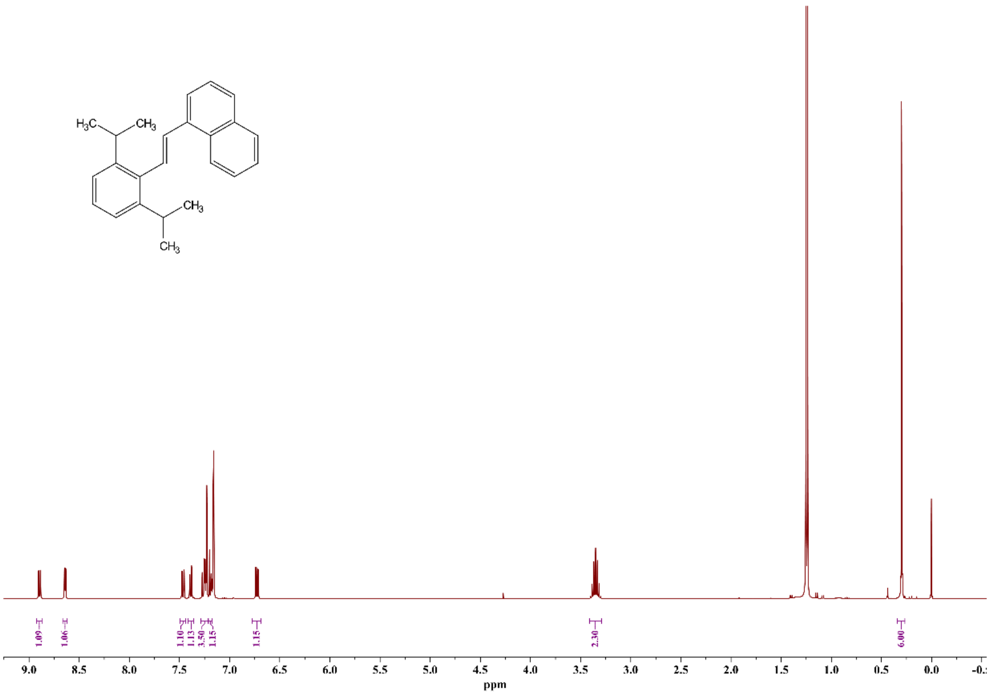

Figure S1. ^{1}H NMR spectrum of L1 in C_6D_6.

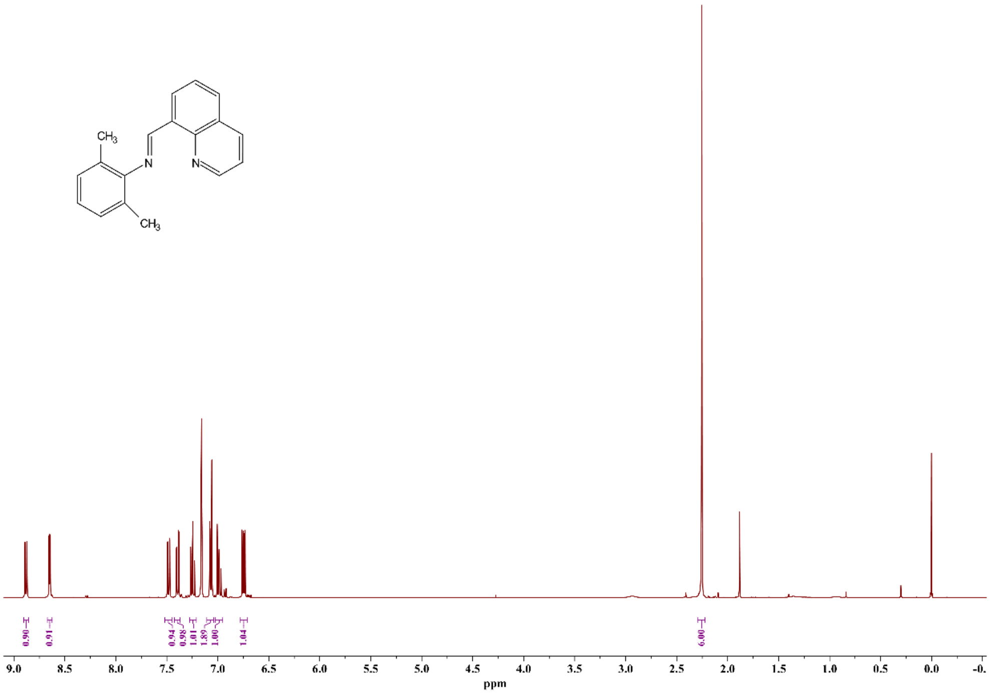

Figure S2. ^{1}H NMR spectrum of L2 in C_6D_6.

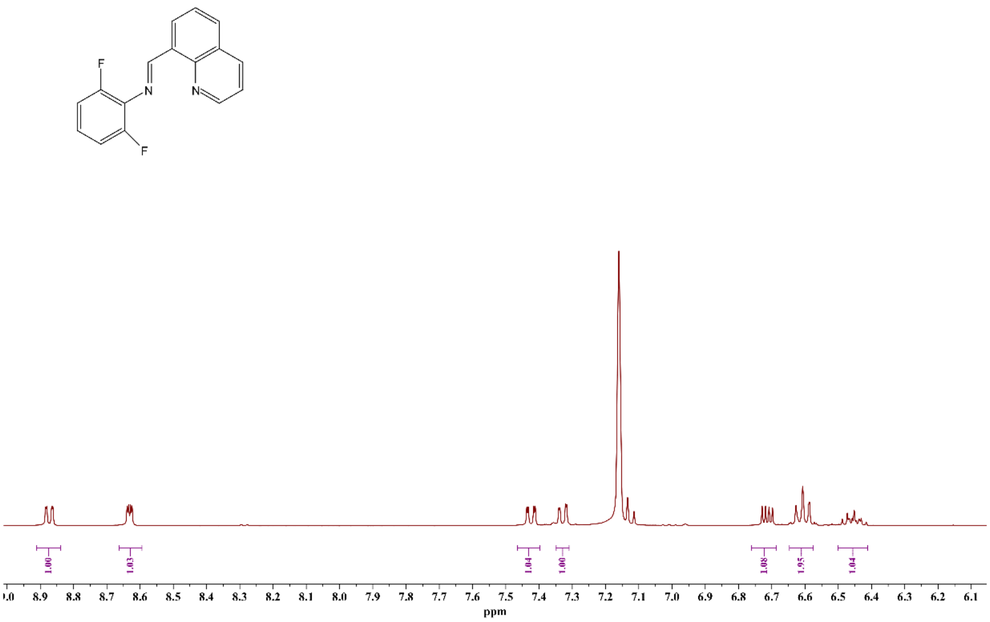

Figure S3. ^{1}H NMR spectrum of L4 in C_6D_6.

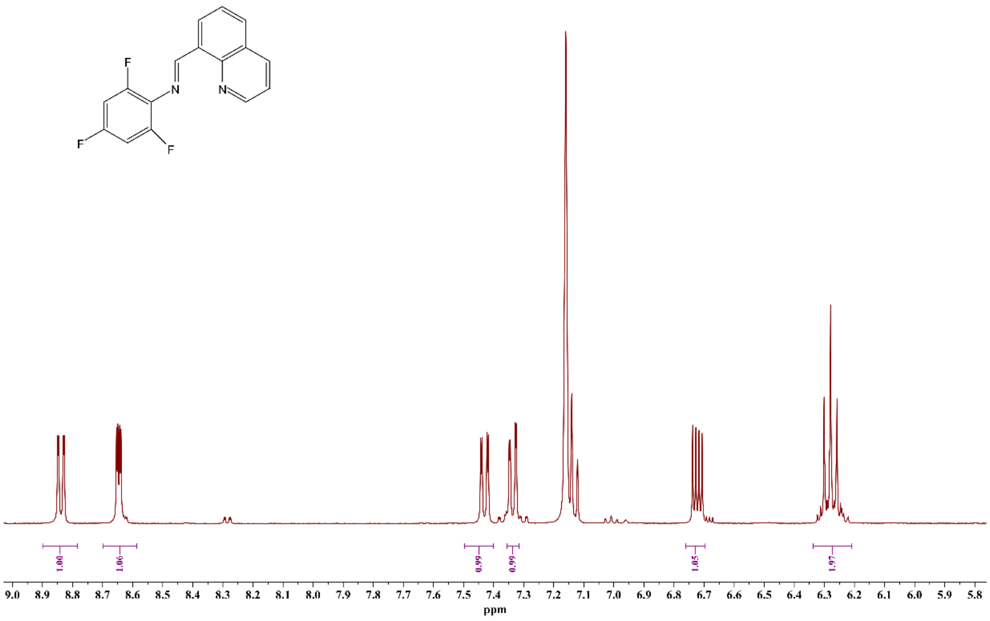

Figure S4. ^{1}H NMR spectrum of L5 in C_6D_6.

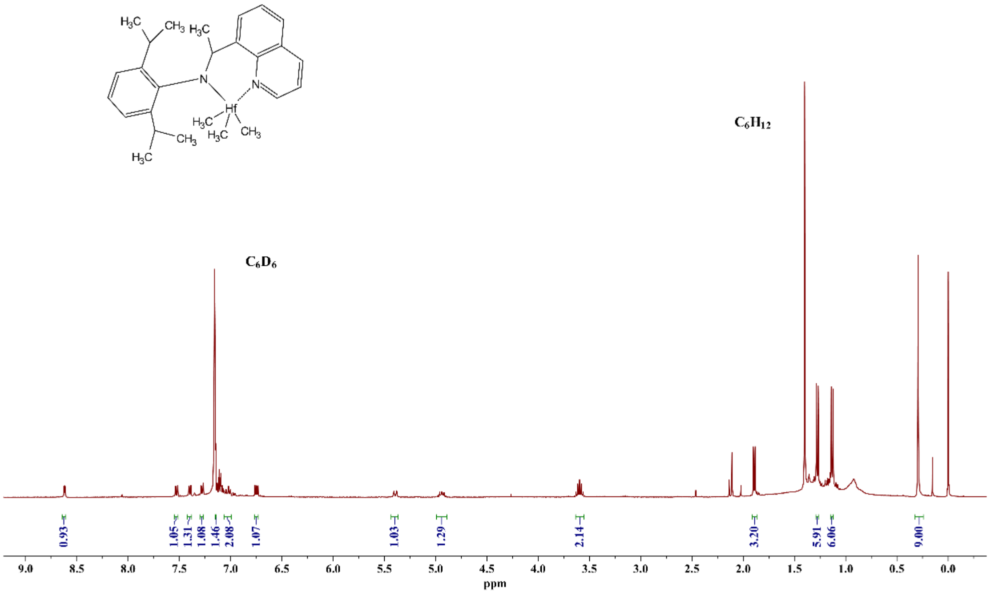

Figure S5. ^{1}H NMR spectrum of Hf-1 in C_6D_6.

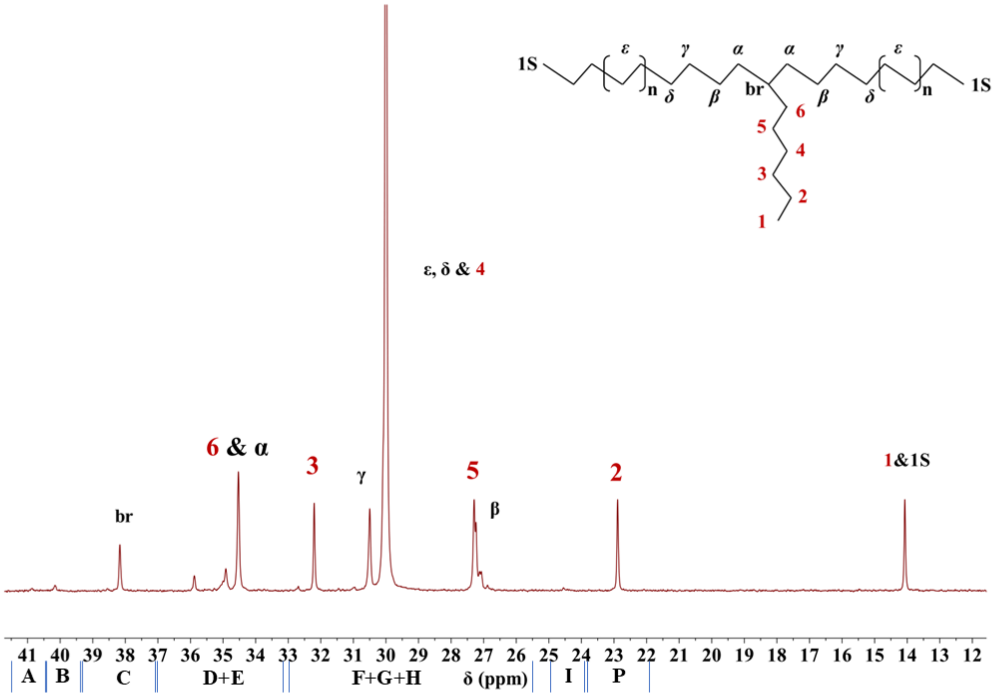

Figure S6. HT-^{13}C NMR spectrum of ethylene/1-octene copolymer by Hf-1.

The 1-Octene contents in the copolymers were calculated according to the literature method.

First, the NMR spectrum is divided into several regions according to the literature method. Next, the peak area of each section is integrated. Using the integration data, the 1-octene content can be calculated.

Table S1. Assignment of chemical shift regions in the NMR spectrum.

	ppm
A	41.5-40.5
B	40.5-39.5
C	39.5-37.0
D	35.8
D+E	36.8-33.2
F+G+H	33.2-25.5
H	28.5-26.5
I	25.0-24.0
P	24.0-22.0

Ethylene/1-Octene copolymers

Mol 1-octene

O1 = *br* carbons: (A + 2C + 2D)/2

O2 = α carbons: [1.5A + 2B +(D + E)-D]/3

O′ = average moles 1-octene = (O1 +O2)/2

Mol ethylene

E′ = {[(F + G +H)-(3A +3B + H + P +I)]/2} + O

Mol% 1-Octene = 100% × O′/(O′ + E′)

Wt% 1-octene = 4 × Mol% 1-Octene/(3 × Mol% 1-Octene+1)

Reference

De Pooter, M.; Smith, P.B.; Dohrer, K.K.; Bennett, K.F.; Meadows, M.D.; Smith, C.G.; Schouwenaars, H.P.; Geerards, R.A. Determination of the composition of common linear low density polyethylene copolymers by ^{13}C-NMR spectroscopy, *J. Appl. Polym. Sci.* **1991,** *42*, 399-408.

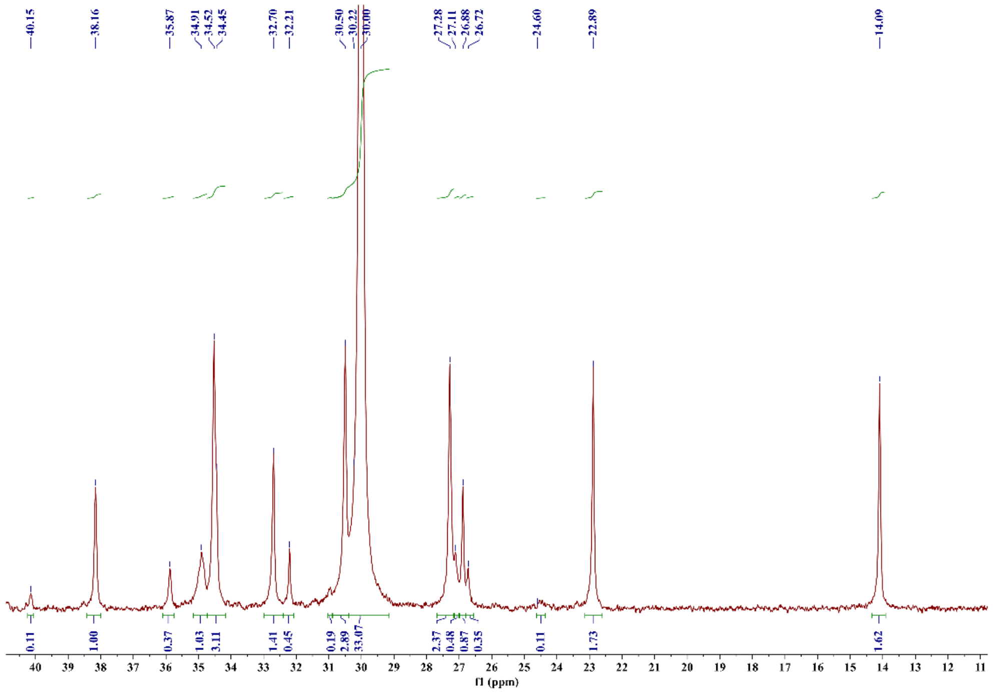

Figure S7. HT-^{13}C NMR spectrum of ethylene/1-octene copolymer by Hf-2.

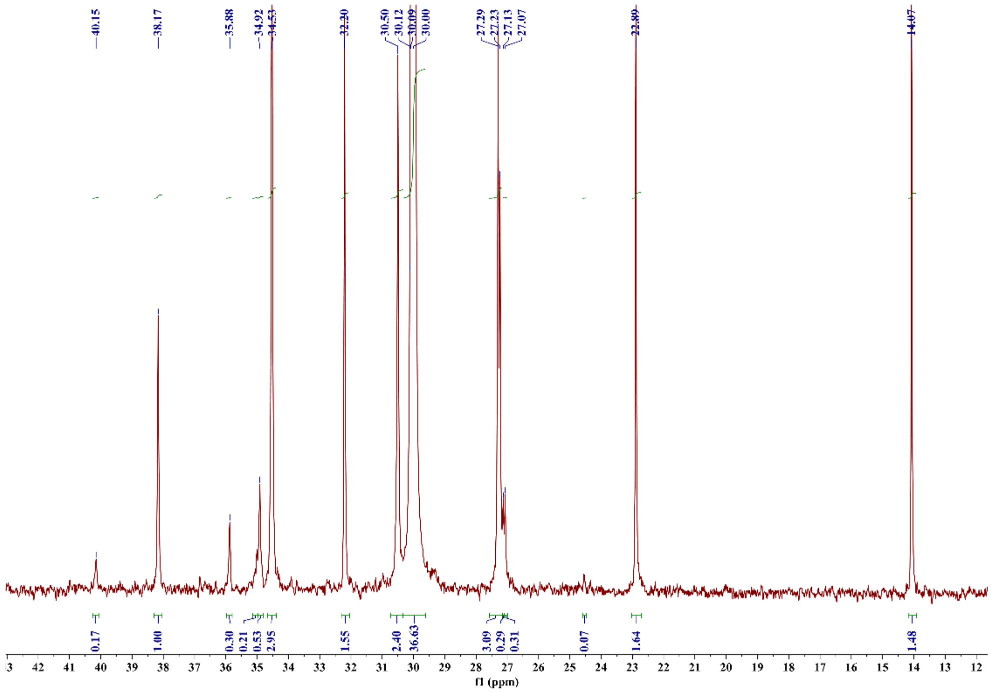

Figure S8. HT-^{13}C NMR spectrum of ethylene/1-octene copolymer by Hf-3.

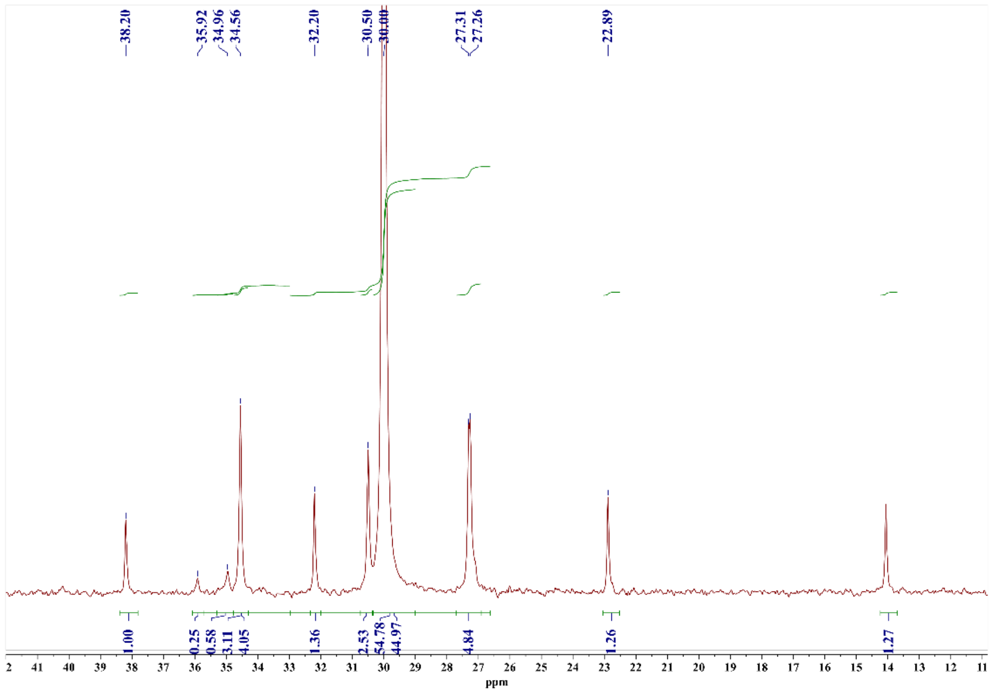

Figure S9. HT-^{13}C NMR spectrum of ethylene/1-octene copolymer by Hf-4.

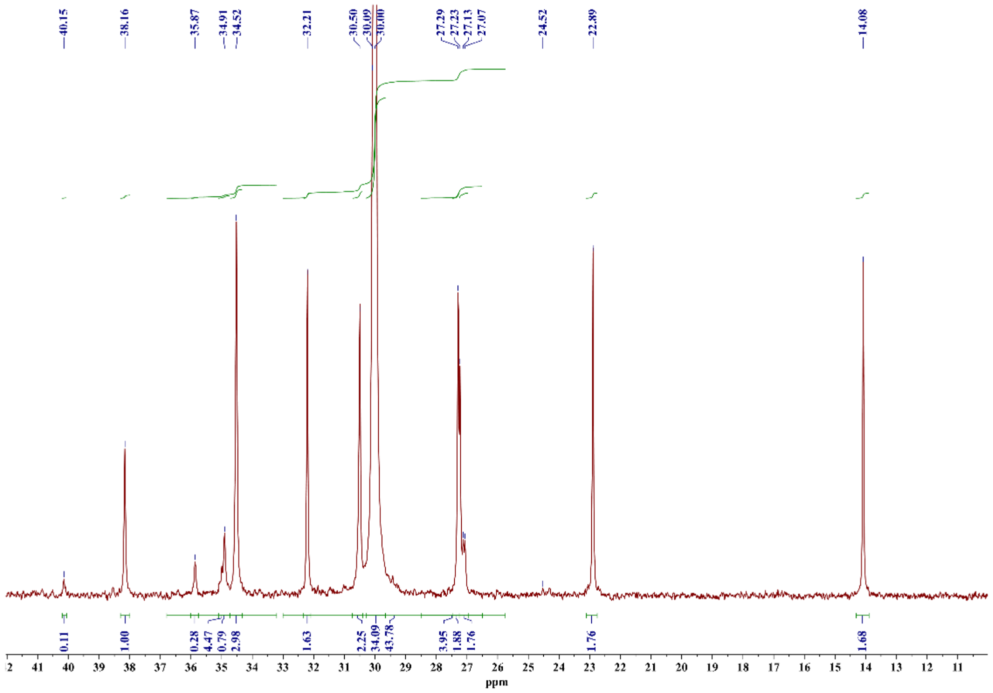

Figure S10. HT-^{13}C NMR spectrum of ethylene/1-octene copolymer by Hf-5.

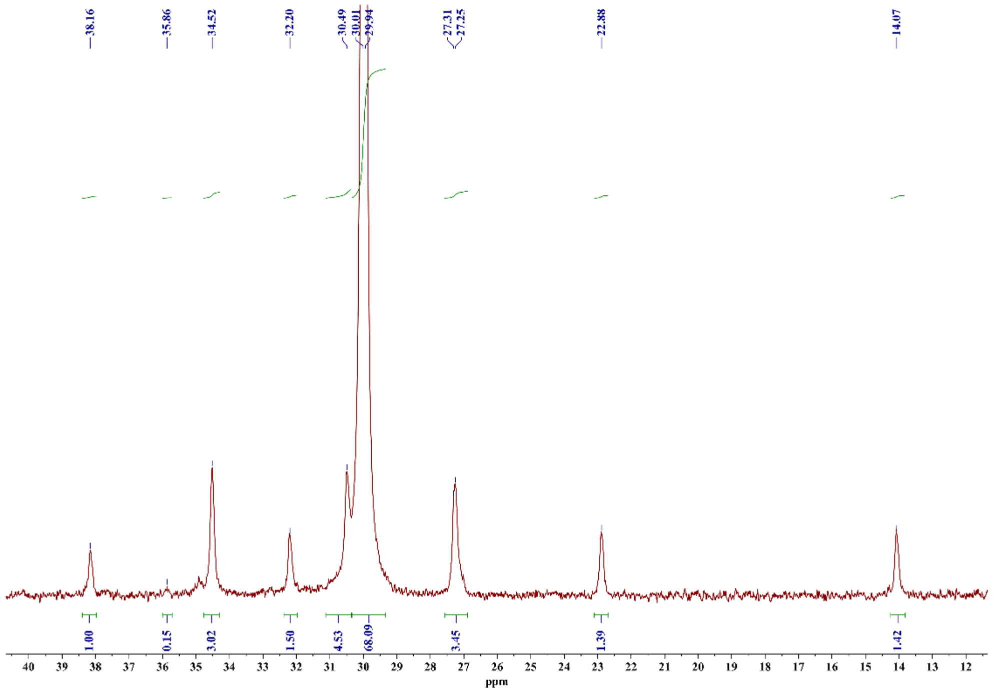

Figure S11. HT-^{13}C NMR spectrum of ethylene/1-octene copolymer by Zr-1.

Table S2. The initial amount of 1-octene feed.

[1-C_8] (M)	[1-C_8] (m/g)	[1-C_8] (V/mL)
0.4	5.3	7.4
0.6	8.2	11.4
0.8	11.3	15.8
